# Prediction of Clearance of Monoclonal and Polyclonal Antibodies and Non-Antibody Proteins in Children: Application of Allometric Scaling

**DOI:** 10.3390/antib9030040

**Published:** 2020-08-05

**Authors:** Iftekhar Mahmood

**Affiliations:** Mahmood Clinical Pharmacology, Consultancy, LLC, 1709, Piccard Dr, Rockville, MD 20850, USA; iftekharmahmood@aol.com; Tel.: +1-301-838-4555

**Keywords:** allometry, children, clearance, therapeutic proteins, monoclonal antibodies, polyclonal antibodies

## Abstract

Allometric scaling can be used for the extrapolation of pharmacokinetic parameters from adults to children. The objective of this study was to predict clearance of therapeutic proteins (monoclonal and polyclonal antibodies and non-antibody proteins) allometrically in preterm neonates to adolescents. There were 13 monoclonal antibodies, seven polyclonal antibodies, and nine therapeutic proteins (non-antibodies) in the study. The clearance of therapeutic proteins was predicted using the age dependent exponents (ADE) model and then compared with the observed clearance values. There were in total 29 therapeutic proteins in this study with 75 observations. The number of observations with ≤30%, ≤50%, and >50% prediction error was 60 (80%), 72 (96%), and 3 (4%), respectively. Overall, the predicted clearance values of therapeutic proteins in children was good. The allometric method proposed in this manuscript can be used to select first-in-pediatric dose of therapeutic proteins in pediatric clinical trials.

## 1. Introduction

During drug development, whether it is in adults or pediatrics, the knowledge of a suitable starting dose is of immense importance. The pharmacokinetics (PK) of a drug is generally known in adults and, for the selection of first-in-pediatric dose, this PK knowledge in adults can be extrapolated to the pediatric population to initiate a clinical trial.

There are three important pharmacokinetic (clearance, volume of distribution, and half-life) parameters which are generally estimated in both adults and pediatrics. Of the three, clearance of a drug is very important because it represents exposure (area under the curve (AUC)) [1] as shown in Equation (1). Therefore, clearance (CL) of a drug can be used for the first-in-children dose selection in order to perform a clinical trial in pediatric population. CL can also be used for dosing in an individual child. Clearance can be calculated from the following Equation (1):

CL = Dose/AUC(1)

where AUC is the area under the curve.

The extrapolation of PK parameters, especially CL for small molecules from adults to children (neonates to adolescents) is well known but for macromolecules (antibodies and non-antibody therapeutic proteins) such extrapolations are not well established [2,3,4,5,6,7]. In a recent study, Mahmood [7] used an allometric approach to predict the clearance of coagulation factors for the initiation of pediatric clinical trials for coagulation factors.

Allometry is the study of size and its consequences and generally used for the prediction of physiological as well as pharmacokinetic parameters [8]. The allometric equation can be generated by either of the following two methods:

I. Using a power function as shown in Equation (2).

Y = aW^b^ (2)

where Y is the parameter of interest (can be a physiological or PK), W is the body weight, “a” and “b” are the coefficient and exponent of the allometric equation, respectively.

II. By linearization of Equation (2) using log transformation as shown in Equation (3).

log Y = log a + b log W(3)

where “a” is the intercept and “b” is the slope.

Allometric scaling is regularly used to predict PK parameters such as clearance, volume of distribution {XE “volume of distribution”}, and half-life {XE “half-life”} from animals to humans (interspecies scaling) [8]. From the same token, the allometric principles can also be applied to scale physiological and PK parameters from preterm neonates to adolescents [2,3,4,5,6,7] from adult data. This approach is of practical value during drug development because it can provide some information for first-in-pediatric dose selection. Allometric scaling based on body weight is easy and simple and can be performed across age groups once PK information of a drug is available from adults.

As clearance is the most important PK parameter, using allometry, it is possible to predict clearance of drugs in children across the age groups from adult drug clearance values [2,3,4,5,6,7]. From these predicted clearance, one can project first-in-pediatric dose to initiate a clinical trial.

The objective of this study was to predict clearance of monoclonal and polyclonal antibodies and non-antibody proteins allometrically from neonates to adolescents using adult clearance values.

## 2. Methods

The values for observed clearance of monoclonal and polyclonal antibodies and non-antibody proteins were obtained from the literature [9,10,11,12,13,14,15,16,17,18,19,20,21,22,23,24,25,26,27,28,29,30,31,32,33,34,35,36,37,38,39,40,41,42,43,44,45,46,47,48,49,50,51] and the predicted clearance values were compared with the observed values for each drug. In this study, there were 13 monoclonal antibodies, seven polyclonal antibodies, and nine therapeutic proteins (non-antibodies). The age stratification was preterm and term neonates (till ≤ 0.25 years), >0.25 to 2 years, >2 to ≤5 years, >5 to ≤12 years, and >12 to <18 years.

Data for basiliximab, canakinumab, gemtuzumab were extracted from the clearance vs. weight or age plots provided by the authors so that age could be stratified to a narrower range. The data extraction was done by digitizeit.

For two polyclonal antibodies Sandoglobulin [35] and Gamimune [36] the clearance values were calculated based on the mean concentration-time data provided by the authors. This was done because it was noted that the clearance values of these two polyclonal antibodies were absolute values rather than body weight adjusted as reported by the authors. The re-calculation of clearance of these two polyclonal antibodies indicated that indeed the clearance values were absolute values rather than body weight adjusted values as reported by the authors.

### 2.1. Prediction of Clearance

The clearance values of therapeutic proteins were predicted using the age dependent exponents (ADE) model. The ADE model is based on variable exponents as a function of age [2,3,4,5,6,7]. Since the allometric exponents widely vary and are data-dependent, in this method, different allometric exponents were used for different age groups and clearance was predicted in a given age group according to Equation (4).

CL = Adult CL × (W_C_/W_70_)^b^ (4)

where the “adult clearance” is the mean adult clearance of a given therapeutic protein obtained from the literature. W_C_ is the weight of a child and W_70_ is the weight of an adult standardized to 70 kg.

Exponent “b” in Equation (4) is age dependent. The exponents used in Equation (4) were 1.2 for preterm and 1.1 for term neonates for age 0–3 months, 1.0, 0.9, and 0.75, >3 months–2 years, >2–5 years, and >5 years, respectively. The exponents selected in the ADE model are based from previous experience, observation, and data analysis. The exponents of the ADE model were previously applied to test the suitability of the exponents in the allometric model across the age group to predict the clearances of small molecule drugs and coagulation factors in children [2,3,4,5,6,7].

### 2.2. Statistical Analysis

Percent error between the observed and predicted values was calculated according to the following equation:

%error = (Pr *edicted* – *observed*) ∗ 100/*observed* (5)

Generally, in the literature, a 2-fold prediction error is considered acceptable. This author, however, considers this magnitude of prediction error too high for any clinical use. Therefore, in this study, a prediction error of ≤50% and ≤30% was considered acceptable error.

## 3. Results

### 3.1. Monoclonal Antibodies

A total of 13 monoclonal antibodies with different age groups were studied. The total number of observations was 33. The observed and predicted clearances in children for monoclonal antibodies are shown in Table 1. The prediction error ranged from 3% to 59%. The number of observations with ≤30%, ≤50%, and >50% was 30 (91%), 32 (97%), and 1 (3%), respectively. Overall, the prediction of clearance for monoclonal antibodies from infants (0.2 year) to adolescents from adult clearance using the ADE model was good.

### 3.2. Polyclonal Antibodies

There were seven polyclonal antibodies (age range = preterm to adolescents) administered either by intravenous or subcutaneous route. The total number of observations was 21. The observed and predicted clearances in children for polyclonal antibodies are shown in Table 2. The prediction error ranged from 0% to 50%. The number of observations with ≤30%, ≤50%, and >50% was 17 (81%), 21 (100%), and 0 (0%), respectively. Overall, the prediction of clearance for polyclonal antibodies from preterm neonates to adolescents from adult clearance using ADE model was good.

### 3.3. Therapeutic Proteins (Non-Antibodies)

There were nine therapeutic proteins with 21 observations. The age ranged from <30 days to adolescents. The observed and predicted clearances in children for therapeutic proteins are shown in Table 3. The prediction error ranged from 1% to 77%. The number of observations with ≤30%, ≤50%, and >50% was 13 (62%), 19 (90%), and 2 (10%), respectively. Overall, the prediction of clearance for therapeutic proteins from neonates to adolescents from adult clearance using ADE model was good.

Overall, the ADE model provided a good prediction of monoclonal and polyclonal antibodies and non-antibody proteins from infants to adolescents. There were 29 macromolecules with 75 observations. The number of observations with ≤30%, ≤50%, and >50% was 60 (80%), 72 (96%), and 3 (4%), respectively (Table 4).

## 4. Discussion

Allometric scaling is a useful tool and can be used to predict PK parameters such as clearance, volume of distribution, and half-life in children from adult data [2,3,4,5,6,7,52,53,54,55]. Allometry has been successfully used for the prediction of clearance in children from adults for small molecules and coagulation factors [2,3,4,5,6,7,53,54,55].

In order to predict PK parameters from adults to children, it is important to consider ontogeny (growth and maturation). In neonates, infants, and toddlers, physiological changes develop very rapidly, and these changes are nonlinear. The physiological parameters of living organisms are allometrically related with body weights or age [8]. Therefore, the use of an allometric model to predict clearance of drugs across the age groups is scientifically and logically based and is not surprising that an allometric model provides a fairly accurate estimate of PK parameters across the age groups.

Considering that the allometric exponents widely vary and are data dependent, and the rapid physiological changes in children are nonlinear, a single exponent cannot be used to describe the clearance versus body weight data across all age groups (neonates to adults) [2,3,4,5,6,55]. Theoretical allometric exponents for clearance (0.75) and volume of distribution (1.0) cannot be used in children generally ≤2 years of age. Determination of the allometric exponents across the age groups (neonates to adults) indicated that the exponents of allometry change with body weight or age [52,56]. This change or difference in allometric exponents as a function of weight or age explains the allometric relationship with ontogeny or maturation for that particular age or body weight and belies the concept of a fixed theoretical exponent across all age groups. This observation led Mahmood to introduce the concept of the age dependent exponent (ADE) model for small and large molecules [2,3,4,5,6,7]. The application of the ADE model led to a substantial improvement in the prediction of drug clearance in children from neonates to adolescents as compared with a single theoretical exponent of 0.75. The theoretical exponent 0.75 substantially over-predicts the clearance of drugs in both preterm and term neonates, infants, and toddlers, hence, should not be used in these age groups.

This study is an attempt to predict clearance of monoclonal and polyclonal antibodies as well as non-antibodies (or therapeutic proteins) allometrically from adult data. In this study, overall, there were 29 therapeutic proteins with 75 observations (age ranging from preterm neonates to adolescents). The prediction error with ≤30%, and ≤50%, was 80% and 96% for 75 observations, respectively (Table 4). Overall, the prediction of the clearance of these macromolecules in children from adult clearance using an ADE model was good.

In this study, one of the issues related to the appropriate application of the exponents of ADE model was the wide range of the age lumped together for many macromolecules. For example, for Cetuximab the age range was 1–12 years (11). Some other examples were Infliximab (0.2–6.25 years) [14], Bivalirudin (1 month to <2 years) [37], and Darbepoetin alfa (1–17 years) [39]. Such study designs over such a wide age range and lumping the PK data together across all age groups is not appropriate since one may not detect the true age dependent PK differences for a given product. Furthermore, an appropriate allometric exponent based on the ADE model could not be applied due the age range and this in some instances might have compromised with the accuracy of the predicted clearance values.

There is paucity of data in preterm and term neonates and infants for macromolecules. In this study, there were two polycolonal antibodies (sandoglobulin and Gamimune) for which data in preterm and term neonates were available. The prediction in term neonates was fairly accurate (<20% prediction error) but in preterm neonates the prediction of clearance was underestimated (less than observed values) by 50% and 47% for sandoglobulin and Gamimune, respectively. Although, the prediction error was within the acceptable range, more data are needed to evaluate the predictive power of the ADE model for antibodies in the preterm and term neonates.

## 5. Conclusions

To the best of the author’s knowledge, this is the first time that an allometric extrapolation has been used to predict clearance of therapeutic proteins (monoclonal and polyclonal antibodies, and non-antibody therapeutic proteins) in children from adult data. It is important to recognize that over the last decade pediatric drug development is on the rise. Therefore, the extrapolation of PK from adults to children is an important step to initiate a pediatric clinical trial. The results of this study indicate that the allometric extrapolation can be used to initiate a pediatric clinical trial with a reasonable initial dose right from the neonates to adolescents. This will save time, effort, and cost from conducting a pediatric clinical trial for rigorous dose finding. Allometric models due to their simplicity and comparable prediction accuracy are more attractive than many other complex models. Many clinical trials fail because the children do not receive an appropriate initial dose (mainly under-dosed due to being over-cautious) because the investigators do not have any clear idea about the first-in-children dose and mostly the dose selection is guess work. It should be, however, noted that the proposed method should only be used for the first-in-dose selection to initiate a pediatric clinical trial and is not in-lieu of a pediatric clinical trial.

## Figures and Tables

**Table 1:** Predicted and observed clearance of monoclonal antibodies in children of different ages.

Age (Years)	Observed CL	Predicted CL	% Error
**Basiliximab, Adult CL = 55 mL/h, Liver Transplantation**			
<2	19	8	−59
3.5	24	18	−26
8.6	28	33	17
13.5	49	43	−13
**Basiliximab, Adult CL = 37 mL/h, Renal Transplantation**			
1–4 y	15	15	−2
6–12 y	19	24	28
14–16 y	30	31	3
**Cetuximab, Adult CL = 46 mL/h, Solid Tumors**			
1–12 y	18	24	35
13–18 y	32	38	20
**Gemtuzumab, Adult CL = 270 mL/h, Refractory or Relapsed Acute Myeloid Leukemia**			
1–5 y	120	87	−27
>5–12 y	180	161	−11
>12–16 y	230	225	−2
**Infliximab, Healthy Adult CL = 9.5 mL/h, children = Kawasaki Disease**			
0.7–3.1 y	1.7	2.0	20
0.2–6.25 y	3.0	2.7	−10
**Natalizumab, Adult CL = 22 mL/h, Crohn Disease**			
11–17 y	18	20	12
**Canakinumab, Adult CL = 9 mL/h, Systemic Juvenile Idiopathic Arthritis (SJIA), SC**			
0.7–1.7 y	1.8	1.3	−27
2.7–4.9 y	4.6	3.2	−30
5.9–11.1 y	7.2	5.9	−17
12–17.2 y	8.3	7.5	−9
**Canakinumab, Adult CL = 9.5 mL/h, Cryopyrin-Associated Periodic Syndrome (CAPS), SC**			
Age not known	2.4	2.7	9
youngest may	5.4	5.1	−6
be 4 years	9.7	6.9	−29
**Urtoxazumab, Healthy Adult CL = 6 mL/h, children = Shiga-Like Toxin (*Escherichia coli*)**			
2.9	1.8	1.9	3
**Tocilizumab, Adult CL = 20.3 mL/h, Polyarticular Juvenile Idiopathic Arthritis**			
20 kg, age not known	8.6	7.9	−8
**Daclizumab, Adult CL = 13 mL/h, Renal Transplant**			
<5 y	5.2	3.2	−38
6–12 y	10.8	6.9	−36
13–17 y	14.5	10.4	−28
**Belacept, Adult CL = 36 mL/h, Kidney Transplant**			
13–17 y	28	32	12
**MEDI8897, Healthy Adults (CL = 65 mL/day) and Healthy Infants, Intramuscular**			
Dose (mg/kg)/age at the time of study, CL is in mL/day			
10 (4.2 months)	4.1	5.3	30
25 (6.7 months)	6.1	6.3	4
50 (7 months)	7.0	6.6	−6
**Bevacizumab,** **Adult CL = 9.8 mL/h, Solid Tumors, Children = CNS Malignancies**			
11–31 months	2.8	2.6	−7
**Palivizumab, Adult CL = 198 mL/Day, Adult and Children = Respiratory Syncytial Virus, Intramuscular**			
12.3 months	11.0	12.7	15

If the route of administration not mentioned, then the antibodies were given by intravenous route.

**Table 2:** Predicted and observed clearance of polyclonal antibodies in children of different ages.

Age (Years)	Observed CL	Predicted CL	% Error
**Panzyga (IV), Adult CL = 101 mL/day, Baseline Uncorrected**			
2–5.9	48	39	−18
6–11.9	50	48	−4
12–16	82	78	−4
**Panzyga (IV), Adult CL = 504 mL/day, Baseline Corrected**			
2–5.9	192	197	3
6–11.9	200	240	20
12–16	504	392	−22
**Gammaplex 10% (IV), Adult CL = 456 mL/day, Baseline Corrected**			
2–5	114	114	0
6–11	178	181	1
12–15	381	367	−4
**GAMUNEX-C (IV), Adult CL = 101 mL/day, Baseline Uncorrected**			
2–5	20	26	31
6–11	44	41	−6
12–16	84	82	−3
**Cuvitru (subcutaneous), Adult CL = 150 mL/day, Baseline Uncorrected**			
2–<5	37	37	1
5–<12	48	59	22
12–16 y	103	117	14
**Hizentra (subcutaneous), Adult CL = 161 mL/day, Baseline Uncorrected**			
6–<12 y	55	74	35
12–<16 y	119	134	13
**Sandoglobulin, Adult CL = 110 mL/day, Baseline Uncorrected**			
Preterm	4.2	2.1	−50
**Gamimune, Adult CL = 110 mL/day, Baseline Uncorrected**			
250 (term)	2.8	3.1	10
500 (term)	4.1	3.3	−18
1000 (preterm)	3.6	1.9	−47

**Table 3:** Predicted and observed clearance of therapeutic proteins (non-antibodies) in children of different ages.

Age (Years)	Observed CL	Predicted CL	% Error
**Bivalirudin, Adult CL = 238 mL/min, Percutaneous Coronary Intervention**			
<30 days	40	9	−77
31 days <2	83	28	−66
2–<6	137	86	−37
6–<16	245	159	−35
**Darbepoetin alfa, Adult CL = 112 mL/h, Chronic Kidney Disease**			
1–17	81	67	−17
<12	55	45	−19
>12	49	64	30
**Erythropoietin, Adult CL = 361 mL/h, End Stage Renal Failure**			
9–12	263	177	−33
>12–16	429	242	−44
**Interleukin 11, Adult CL = 26 L/h, Solid Tumors or Lymphoma**			
>1–3	4.9	4.1	−17
>3–<13	13.2	12.5	−5
>13–<17	23.8	20.4	−14
**Drotrecogin alfa (activated), Adult CL = 38 L/h, Chronic Kidney Disease**			
<1 year	3.4	3.3	−4
1–8 year	12	18	46
**Glulisine, SC, Adult CL = 53 mL/h, Type 1 Diabetes**			
5–11 year	45	35	−23
11–17 year	65	50	−24
**Regular Human Insulin, SC, Adult CL = 55 mL/h, Type 1 Diabetes**			
5–11 year	65	36	−44
11–17 year	63	51	−18
**Growth Hormone (LB03002), Adult CL = 25 L/h, GH Deficiency**			
7 years	9.1	11.5	27
**Enfuvirtide, Adult CL = 1310 mL/h, HIV-1-Infected, SC**			
>5–<12 year	666	640	−3
>12–<17 year	970	960	−1

**Table 4:** Summary statistics of the analysis by the age dependent exponents (ADE) model.

Description	Monoclonal	Polyclonal	Non-Antibodies
# of drugs	13	7	9
# of observations	33	21	21
≤30% PE	30 (91%)	17 (81%)	13 (62%)
≤50% PE	32 (97%)	21 (100%)	19 (90%)
≥50% PE	1 (3%)	0 (0%)	2 (10%)
# All drugs	29		
# of observations	75		
≤30% PE	60 (80%)		
≤50% PE	72 (96%)		
≥50% PE	3 (4%)		

PE = percent error; numbers in brackets are percent of total.
